# Quality and Reliability of TikTok Videos on Uterine Fibroid Embolizations: An Observational Analysis of Internationally Trending Social Media Videos

**DOI:** 10.7759/cureus.89372

**Published:** 2025-08-04

**Authors:** Shreya Katwala, Ashna Chali, Michelle Wu, Felix Yang, Mira Malavia, Trung Pham

**Affiliations:** 1 Internal Medicine, University of Missouri Kansas City School of Medicine, Kansas City, USA; 2 Anesthesia, Critical Care, and Pain Medicine, University of Missouri Kansas City School of Medicine, Kansas City, USA; 3 Interventional Radiology, University of Missouri Kansas City School of Medicine, Kansas City, USA

**Keywords:** evidence-based clinical practice, medical misinformation, multiple uterine fibroids, patient education, social media analytics, tiktok, uterine fibroid embolization, video content analysis

## Abstract

Introduction: TikTok has emerged as a popular platform for sharing medical insights, but concerns exist regarding disseminating inaccurate information on medical conditions, potentially harming patient care. This study aims to evaluate the quality and reliability of TikTok videos on uterine fibroid embolization (UFE). It also examines how video engagement and content quality vary based on the uploader type and video style.

Methodology: We selected the top 100 TikTok videos on UFEs based on the number of likes since March 2024. These videos were identified using the hashtag "#UFE." Videos were categorized based on several factors, including the number of likes, comments, shares, upload date, uploader's background (academic, non-healthcare professionals (non-HCPs), non-physician, non-radiologist, and radiologist), and type of content (anecdotal, educational, and procedural). The strength of treatment recommendations was assessed for all videos using the DISCERN instrument (16 to 80), which assesses reliability of treatment information, and the Patient Education Materials Assessment Tool for Audiovisual Materials (PEMAT-A/V), which assesses understandability and actionability via a percentage score. Descriptive and ANOVA analyses were conducted with a statistical significance set at p < 0.05.

Results: Of the top 100 TikTok videos related to UFE, 99 were in English and one was in Spanish, which was excluded from our study. A total of 12 videos (12.12%) were uploaded by radiologists, while the majority (66, 66.67%) were created by non-HCPs. Non-radiologist physicians garnered the highest average engagement and quality scores, including the highest DISCERN score (41.07). Academic institutions achieved the highest PEMAT understandability score (80.47%). Non-HCPs ranked second in engagement but had the second-lowest PEMAT understandability score (65.96%), just above non-physician HCPs, who had the lowest score (64.68%). Educational videos (35, 35.35%) outperformed anecdotal ones (59, 59.59%) in quality, achieving higher DISCERN (40.57) and PEMAT understandability (68.85%) scores. While DISCERN scores did not significantly differ by video aim, both PEMAT understandability (p = 0.01763) and DISCERN (p = 0.00166) scores showed significant differences based on the uploader type.

Discussion: Our analysis of the top 100 TikTok videos on UFE reveals a landscape dominated by non-physician contributors, with only 12 videos created by radiologists. Despite this, content from non-radiologist physicians garnered the highest engagement and exhibited the highest quality, underscoring the influence of credible medical voices on social media. While anecdotal content prevails, educational videos achieved better quality scores, highlighting the value of evidence-based communication. These findings suggest a critical opportunity for radiologists and other physicians to enhance patient education, counter misinformation, and leverage TikTok as a low-cost, high-impact platform for healthcare communication. However, limitations include a narrow focus on highly liked content and exclusion of TikTok’s algorithmic influence.

## Introduction

Social media and online sources have become integral to modern healthcare, offering a wealth of benefits to both patients and healthcare providers. These platforms foster support for communities by facilitating connections among patients who share similar health experiences. Additionally, they provide a readily accessible source of health information, serving as a resource for patients seeking guidance when they are unable to consult a healthcare professional immediately. Over 80% of US State Health Departments have embraced social media platforms to enhance accessibility for the general public, allowing individuals to stay informed about their health and connected at all times [1].

The increasing accessibility of health-related information has empowered patients globally to conduct research into their medical concerns. Extensive survey data pooling thousands of Internet users demonstrated that over 40% of people use the Internet to research health information [2]. This influx of readily available health information online offers many advantages, including alternative options, insights, and a broader knowledge base, enhancing patient engagement both on a personal level and in the clinic [3]. However, the rapid proliferation and dissemination of health information online also raise concerns about misinformation. It was found that from 2006 to 2017, 126,000 news stories were tweeted by three million people more than 4.5 million times. Of these, false stories were more likely to spread further, faster, and deeper [4]. Despite this challenge, the influence of social media on patient decision-making and engagement remains undeniably powerful.

TikTok, a leading social media platform, has become a prominent source of health-related content, particularly in the form of short videos. With over a billion users globally as of 2020, and 41% of its user base aged between 16 and 24, TikTok’s short-form, hashtag-driven content makes it an accessible tool for exploring a vast array of topics [5,6]. Health-related hashtags such as “medicine” and “doctor” have garnered 1.4 billion and 6.7 billion views, respectively [6]. TikTok’s vast audience, particularly among younger populations, presents an opportunity for healthcare professionals to disseminate important health information to the public, potentially improving patient education and awareness. However, due to the lack of official scientific oversight, it is possible for both intentional and unintentional misinformation to gain a wide reach on TikTok and similar platforms [5]. Additionally, the presence of financial conflicts of interest can be a complicating factor in the distribution of health information on this platform and can also influence public trust in physicians and the healthcare system [7].

Uterine fibroid embolization (UFE) is a minimally invasive alternative for those suffering from symptomatic uterine fibroids. Unlike traditional surgical approaches, UFE involves a catheter guided by imaging to deliver embolic agents to the arteries that feed the fibroids, cutting off their blood supply. This targeted embolization causes the fibroids to shrink, offering relief from symptoms like heavy menstrual bleeding, pelvic pain, and frequent urination. With its shorter recovery time and reduced risk compared to surgery, UFE presents a compelling option for women seeking to regain their quality of life without the drawbacks of invasive surgery [8]. However, potential disadvantages of this procedure include negative effects on fecundity and pregnancy as well as the potential for recurrence [9]. Traditional techniques for treating uterine fibroids include hysterectomy, or surgical removal of the uterus, and myomectomy, or removal of the fibroids while leaving the uterus intact. Both surgeries are invasive and require general anesthesia, with longer recovery times compared to minimally invasive options like UFE [10]. Due to these benefits, UFE is becoming more popular and is a viable target of exploration in regard to the role of social media in distributing health information [11].

This study analyzes the efficacy of TikTok as a platform for disseminating high-quality health information to the public, specifically regarding UFE. Previous research has explored the quality of TikTok videos aiming to share information about knee osteoarthritis and intrauterine device (IUD) placement, as well as the quality of YouTube videos about UFE [12-14]. This study aims to evaluate the quality and reliability of TikTok videos on UFE. It also examines how video engagement and content quality vary based on the uploader type and video style.

## Materials and methods

**Figure 1:**
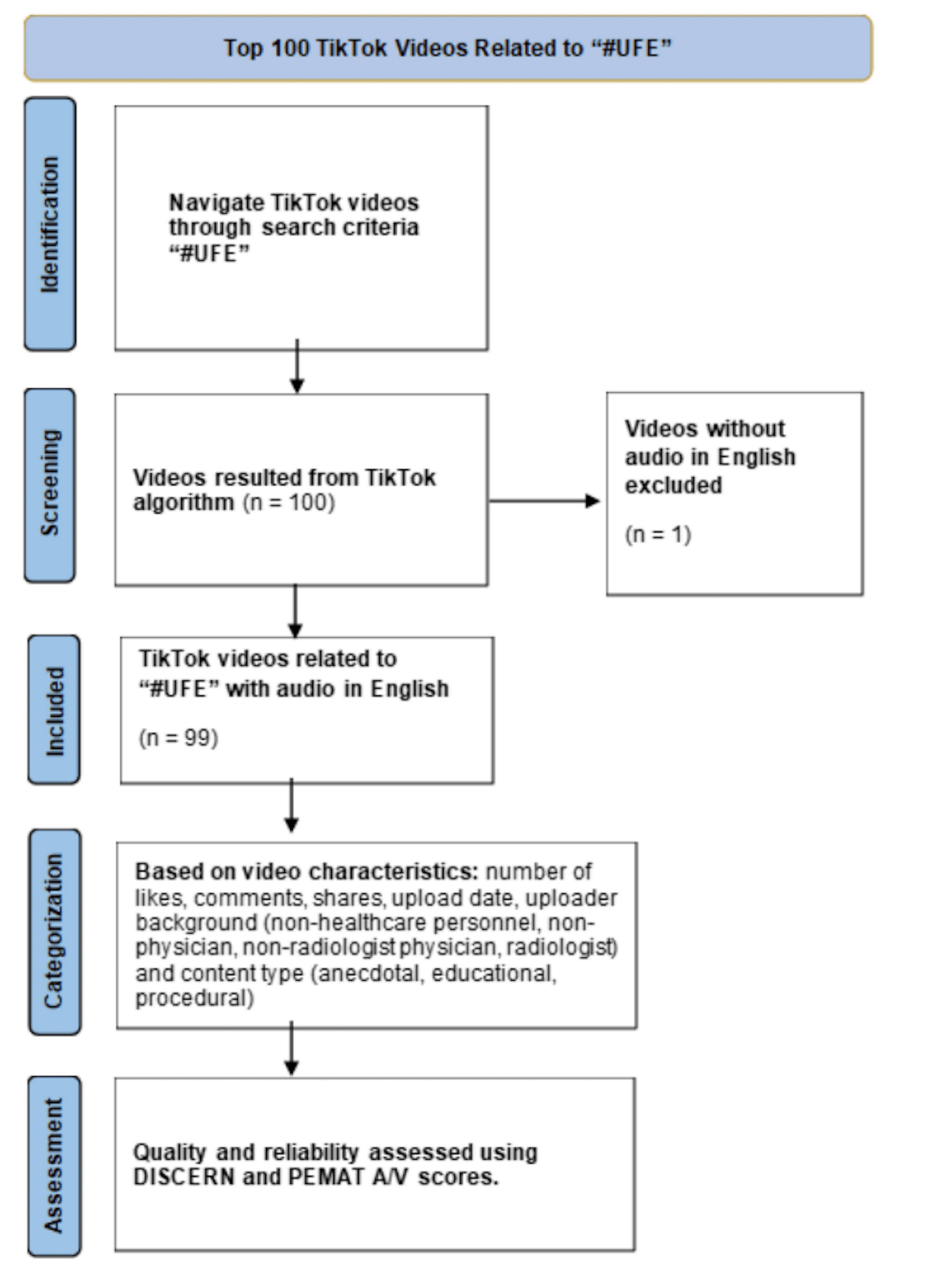
Top 100 TikTok Videos Related to "#UFE" Flowchart of observational analysis for TikTok videos related to uterine fibroid embolization (UFE)

This observational study involved the evaluation of TikTok posts up until March 31, 2024. The TikTok search engine was utilized to identify the top 100 publicly available videos associated with the hashtag "#UFE" (Figure 1). To mitigate potential bias from the TikTok algorithm, a new account was created specifically for this study. This ensured that the videos selected did not reflect a single author’s preferences; however, the authors of this study were not blinded to the identity of the video uploaders. Additionally, we assumed that these 100 videos exerted the greatest influence on the audience due to their viral nature. Among these top videos, 99 were in English, and one was in Spanish; the latter was excluded from our analysis.

Next, each video was systematically categorized in Microsoft Excel (Microsoft Corp., Redmond, WA, US) based on the following variables: number of likes, comments, shares, upload date, uploader background (radiologist, other physician, non-physician healthcare professional, non-healthcare individual, or commercial entity), and content type (educational, procedural, and anecdotal/personal story). To evaluate the quality of health information presented, we applied two validated assessment tools: (1) the DISCERN (16-80) instrument to assess the reliability and quality of treatment information and (2) the Patient Education Materials Assessment Tool for Audiovisual Materials (PEMAT-A/V) to evaluate content understandability and actionability, reported as a percentage score. The DISCERN score was a series of 16 questions with 1-5 points associated with it, and the summation was reported. DISCERN scores were divided into very poor (16-29), poor (30-40), fair (41-51), good (52-63), and excellent (64-80) [15]. For PEMAT scores, higher values indicated greater understandability and actionability. Descriptive statistics and ANOVA single-factor analyses were performed to characterize the dataset and explore associations between video characteristics, quality scores, and user engagement metrics. The statistical significance was set at p < 0.05.

## Results

Of the top 100 TikTok videos identified using the hashtag “#UFE,” 99 were in English and included in the final analysis. Of those, 66 (66.66%) were uploaded by non-healthcare professionals (non-HCPs), while 12 (12.12%) were uploaded by radiologists. Other uploader types included non-radiologist physicians (15 (15.15%)), academic institutions (3 (3.03%)), and non-physician healthcare professionals (3 (3.03%)) (Table 1).

**Table 1:** Characteristics of the Top 100 TikTok Videos on Uterine Fibroid Embolization by Uploader Type p-values reflect comparisons of metrics across uploader types. p < 0.05 indicates statistical significance. * indicates statistically significant p-values. HCP: non-healthcare professional

Uploader type	Number of videos	Average number of likes	Average number of comments	Average number of shares	Average DISCERN score	DISCERN p-value	Average PEMAT understandability score	PEMAT understandability p-value	Average PEMAT actionability score	PEMAT actionability p-value
Academic	3	58	4	11	40.33 ± 14.22	-	0.8048 ± 0.1692	-	0.3333 ± 0.5774	-
Radiologist	12	28.5	3	4	35.67 ± 4.355	-	0.7046 ± 0.1254	-	0.1389 ± 0.3321	-
Non-radiologist physician	15	1,262	134	134.5	41.07 ± 7.166	-	0.6997 ± 0.121	-	0.1778 ± 0.3572	-
Non-physician HCP	3	46	2	2.5	24.33 ± 1.1547	-	0.6469 ± 0.1069	-	0.3333 ± 0.5774	-
Non-HCP	66	125.5	72.5	60.5	37.97 ± 8.274	-	0.6596 ± 0.1063	-	0.2374 ± 0.3279	-
p-value	-	-	-	-	-	0.00166*	-	0.01763*		0.72995

Non-radiologist physicians garnered the highest average likes (1,262) and shares (134.5) per video, with the highest average DISCERN score of 41.07. Non-HCPs followed with the second-highest average number of likes (125.5) and shares (60.5), accompanied by a DISCERN score of 37.97, slightly below that of academic institutions (40.33). Academic institutions attained the highest PEMAT understandability score (80.47%), followed by radiologists (70.46%), while non-physician HCPs recorded the lowest average PEMAT understandability score (64.68%). Academic institutions achieved the highest average PEMAT actionability score (33.33%), whereas radiologists registered the lowest average PEMAT actionability score (13.89%).

Regarding video content, most of the top videos primarily aimed for anecdotal storytelling (59, 59.59%), with this category yielding the lowest average DISCERN score of 36.86. Conversely, videos with an educational focus constituted 35 (35.35%) of the top videos and obtained the highest DISCERN score within the video aim categories (40.57). Videos with a procedural aim also garnered the highest average PEMAT understandability (72.62%), and anecdotal videos had the highest actionability (28.25%) scores (Table 2).

**Table 2:** Characteristics of the Top 100 TikTok Videos on Uterine Fibroid Embolization by Video Aim p-values reflect comparisons of metrics across video aims. p < 0.05 indicates statistical significance. * indicates statistically significant p-values.

Video aim	Number of videos	Average number of likes	Average number of comments	Average number of shares	Average DISCERN score	DISCERN p-value	Average PEMAT understandability score	PEMAT understandability p-value	Average PEMAT actionability score (%)	PEMAT actionability p-value
Anecdotal	59	116.05	27.76	8.32	36.86 ± 8.87	-	0.6629 ± 0.1127	-	0.2825 ± 0.3474	-
Educational	35	1,961.4	64.69	353.4	40.57 ± 8.19	-	0.6885 ± 0.1119	-	0.1524 ± 0.3462	-
Procedural	5	150	20.8	32.8	37.6 ± 10.53	-	0.7262 ± 0.1496	-	0 ± 0	-
p-value	-	-	-	-	-	0.1392	-	0.105	-	0.00648*

Statistical analysis revealed a significant difference in DISCERN scores across uploader types (p = 0.00166), as well as in PEMAT understandability scores (p = 0.01763). However, no significant difference was found in PEMAT actionability scores among uploader types (p = 0.7299). Similarly, DISCERN (p = 0.1391) and PEMAT understandability (p = 0.1049) scores did not significantly differ by video aim. Notably, PEMAT actionability scores did show a significant difference based on video aim (p = 0.00648).

## Discussion

Social media has become an increasingly influential force in healthcare communication, offering new avenues for patient education, outreach, and engagement. Both medical professionals and individuals seeking healthcare information actively engage with these platforms, leveraging their swift and accessible nature to exchange and acquire knowledge on health-related matters [16,17]. Nearly 80% of Internet users resort to online platforms for healthcare inquiries, with social media serving as a primary avenue for patients to gather insights about their conditions, treatments, and healthcare providers [16,17]. Moreover, studies have underscored the efficacy of social media in augmenting knowledge, shaping attitudes, and fostering self-care practices, as evidenced by successful initiatives targeting patients with conditions like diabetes [18]. These platforms both facilitate the dissemination of health-related information and provide a space for individuals to pose queries and raise awareness about diverse medical diagnoses. The healthcare sector is witnessing a surge in TikTok usage, making it a notable platform for medical content during the 2020s [6].

Previous research has shown that social media can enhance patient knowledge, influence behavior, and support self-management of chronic diseases [19-21]. These platforms facilitate interactive communication, allowing users to both access and contribute to discussions about health, which has proven effective in raising awareness and fostering community support [22]. However, despite their growing popularity, radiologists and other medical specialists remain underrepresented on these platforms. In a study by Lovett et al., only 5% of TikTok videos tagged with “#radiology” were created by radiologists, though these comprised most of the clinically informative content [23]. This discovery emphasizes an unexplored potential for utilizing this platform to improve radiology patient education and communication. Similar outcomes were reflected in our study, as of the top 100 videos, only 27 were uploaded by physicians, with only 12 being uploaded by radiologists.

Despite the relatively small number of physician-created videos, their influence was still impactful. Non-radiologist physicians produced content with the highest average number of likes (1,262), shares (134.5), and comments (134), as well as the highest average DISCERN score (41.07) and the third-highest PEMAT understandability score (69.96%). Academic institutions achieved the highest PEMAT actionability score (33.33%), though actionability scores across all uploader types were relatively low. Radiologists recorded the lowest actionability score at 13.89%. This low score may be due to a variety of factors including a lack of patient-oriented language or detailing specific action steps. In contrast, 66 of the videos were created by non-HCPs, who, despite achieving high engagement, had the lowest PEMAT understandability scores (65.96%), indicating that non-HCP content may be more challenging for audiences to comprehend. This presents an opportunity for radiologists and non-radiologist physicians to engage on TikTok and reshape the discourse surrounding UFEs. It offers a chance to dispel misconceptions and potentially steer patients toward treatments aligned with approved guidelines. If harnessed effectively, TikTok holds the potential to boost patient engagement, facilitate remote follow-up visits, and foster surgeon education through real-time interaction, all at minimal additional costs.

Our analysis of the top TikTok videos related to UFEs reveals the prominent role of non-HCPs in content creation, with non-radiologist physicians leading in both engagement and quality. While anecdotal storytelling is common on the platform, videos with an educational focus achieved higher quality scores (DISCERN: 40.57) and better PEMAT understandability, reinforcing the value of evidence-based content. The significant p-values for DISCERN (p = 0.00166) and PEMAT understandability (p = 0.01763) highlight meaningful differences in content quality based on uploader type, demonstrating the importance of professional involvement to ensure accurate and accessible information. Additionally, the significant p-value for PEMAT actionability (p = 0.00648) emphasizes the need for content that encourages actionable health behaviors, suggesting that targeted educational videos could enhance the effectiveness of social media as a tool for patient education.

However, limitations exist. This study examined only the 100 most-liked TikTok videos using the hashtag “#UFE,” one of which was excluded, potentially overlooking relevant content that was less popular but of higher quality. Additionally, the influence of TikTok’s proprietary algorithm on video visibility and engagement was not evaluated. Lastly, although the DISCERN and PEMAT tools are validated, they were originally designed for written or long-form audiovisual content, which may not fully capture the nuances of short-form video typical of TikTok.

Nonetheless, this study emphasizes a critical opportunity for radiologists and other healthcare providers to engage with patients via social media. Given that physician-created content consistently achieves higher engagement and quality scores, as per our study, increasing the presence of interventional radiologists on platforms like TikTok could counter misinformation and promote evidence-based care. Social media can also serve as a cost-effective tool for improving patient education, fostering trust, and encouraging informed decision-making, particularly for complex procedures such as UFE.

In light of these findings, we recommend that interventional radiologists consider adopting social media strategies and advocate for professional societies to support such initiatives. By providing high-quality, accessible content, radiologists can play a pivotal role in shaping online health narratives and enhancing public understanding of minimally invasive treatment options. However, interventional radiologists should caution their patients that information on UFEs found on TikTok may not adhere to medical guidelines.

## Conclusions

As social media continues to redefine the landscape of health communication, platforms like TikTok are increasingly shaping patient perceptions, treatment preferences, and healthcare decision-making. This study highlights a significant gap between the volume of TikTok content related to UFE and the credibility of its sources. In our study, the overwhelming majority of videos were created by non-HCPs, while physician-generated content remained underrepresented, despite demonstrating higher quality and greater engagement. By recognizing the influence and accessibility of TikTok, interventional radiologists can engage in this digital space, guiding patients toward evidence-based treatments and fostering a better understanding of procedures such as UFEs. TikTok is a social media platform that warrants consideration as a tool for patient education, as long as it is used with discretion.

Future research should explore broader and more representative samples, assess the impact of social media algorithms, and evaluate longitudinal outcomes related to patient knowledge and treatment decisions. As the boundaries between digital media and medicine continue to blur, embracing social media literacy and engagement as core competencies for healthcare providers may become essential to patient-centered care.
